# Growth Without GH: A Case Series and Literature Review

**DOI:** 10.3390/jcm14248957

**Published:** 2025-12-18

**Authors:** Stefana Catalina Bilha, Cristina Preda, Letitia Leustean, Nada Akad, Anca Matei, Maria-Christina Ungureanu

**Affiliations:** 1 Endocrinology Department, Grigore T. Popa University of Medicine and Pharmacy, 700115 Iasi, Romania; stefana.bilha@umfiasi.ro (S.C.B.); cristina.preda@umfiasi.ro (C.P.); akad_nada@d.umfiasi.ro (N.A.); anca.matei@umfiasi.ro (A.M.); maria.ungureanu@umfiasi.ro (M.-C.U.); 2 Endocrinology Department, Clinical Emergency Hospital “Sf. Spiridon”, 700111 Iasi, Romania

**Keywords:** growth without growth hormone, GH deficiency, linear growth, insulin resistance, obesity

## Abstract

Linear growth is traditionally attributed to the growth hormone (GH)/insulin-like growth factor-1 (IGF-1) axis, yet “growth without GH” is documented. We report five patients with severe GH deficiency—one congenital and four acquired, who reached normal or tall stature despite persistently low IGF-1. All patients had obesity and metabolic complications (insulin resistance, dyslipidemia, and/or fatty liver). Catch-up or sustained growth occurred before or independent of sex-steroid replacement in most cases. One patient with lifelong hypogonadism showed slow, prolonged growth with delayed epiphyseal fusion. Three patients also received recombinant human GH (rhGH), without a significant impact on overall growth velocity, but with favorable metabolic outcomes. Findings support multifactorial drivers of linear growth beyond the GH/IGF-1 pathway. Likely contributors include insulin signaling associated with adiposity, permissive thyroid hormone action, local growth-plate paracrine pathways, and, in hypogonadism, delayed epiphyseal closure. Genetic modifiers that enhance chondrogenesis or delay growth-plate fusion may contribute. We also reviewed the published literature on “growth without GH,” integrating single-case reports and series to contextualize these mechanisms and outcomes. In conclusion, profound GH deficiency does not preclude near-normal or accelerated growth. In “growth without GH,” therapeutic priorities should pivot from stature to cardiometabolic risk reduction. rhGH may be considered to improve metabolism when individualized and closely monitored, recognizing that height velocity is often adequate. Notably, rhGH consistently improved lipid profiles and steatohepatitis in two patients, suggesting a primarily metabolic benefit. Lifelong follow-up from childhood into adulthood is essential.

## 1. Introduction

Height gain depends on the growth plate’s rate of cartilage cell formation. Growth hormone (GH) secreted by the anterior pituitary gland plays a crucial role in this regulatory framework, as it directly affects various tissues and stimulates the production of insulin-like growth factor-1 (IGF-1) in the liver, promoting linear bone growth [1].

Besides the GH/IGF-1 axis, multiple other factors regulate growth: sex steroids, thyroid hormones, insulin, prolactin, and genetic variants influencing growth plate chondrogenesis all contribute to height and somatic development [2,3,4].

“Growth without GH” has been reported in certain cases, particularly in patients with tumors affecting the hypothalamic-pituitary region, after surgery or other therapies for brain tumors. In such instances, normal height development can occur after tumor resection despite low or absent GH levels, as these patients often experience various metabolic abnormalities, including obesity, dyslipidemia, and fatty liver [5]. Moreover, in certain cases of multiple pituitary deficiencies, whether idiopathic, due to PROP1 mutations, or pituitary stalk interruption syndrome, individuals have experienced normal linear growth despite GH deficiency and the absence of obesity or hyperinsulinemia [5,6,7,8,9,10,11]. Nonetheless, tall stature without GH was reported in prolonged hypoestrogenism, such as males with aromatase deficiency [12], congenital or acquired hypogonadism [13,14], and in rare androgen-biosynthesis defects (e.g., 17,20-lyase/CYP17A1 deficiency) [15].

We report five GH-deficient patients who achieved normal or even tall stature and present a focused literature review of similar cases to contextualize mechanisms (e.g., IGF/insulin signaling, delayed epiphyseal fusion due to prolonged hypogonadism, genetic regulation, and local growth-plate paracrine signals), clinical evolution, and management considerations. We also discuss the role of human GH (hGH) therapy primarily for metabolic and body-composition improvement in the “growth without GH” phenotype.

## 2. Case Report

### 2.1. Case 1

A 43-year-old male with a family history of diabetes mellitus (DM) in both his parents and one sister, with a personal history of type 2 DM and arterial hypertension diagnosed 3 years prior, is referred to our Endocrinology Department for lack of secondary sexual characteristics.

On clinical examination, the patient was 182.5 cm tall (+0.83 standard deviations (SDS)), he weighed 81 kg with a body mass index (BMI) of 24.5 kg/m^2^, arm span was 181 cm; he associated orthostatic hypotension (sitting BP 150/80 mm Hg and standing BP 123/79 mm Hg), bradycardia (heart rate 42 b/min), pectus excavatum, high arched palate, kyphosis, pale and dry skin, absence of sexual-dependent body hair and undescended testes.

Hormonal work-up confirmed hypopituitarism with impairment of thyroid (Thyroid Stimulating Hormone (TSH) = 2 uUI/mL, normal range (NR) 0.4–4, free T4 (FT4) = 0.41 ng/dL, NR 0.89–1.76), adrenal (8 am cortisol = 3 µg/dL, NR 5–25), gonadal (Luteinizing Hormone (LH) < 0.1 mUI/mL, NR 2–11, Follicle-Stimulating Hormone (FSH) = 0.16 mUI/mL, NR 2–11, testosterone < 0.087 nmol/L, NR 8.64–2.9, estradiol < 5 pg/mL, NR 7–42) and somatotroph axes (with very low IGF-1 levels of 19.1 ng/mL, NR 101–267). Anti-Mullerian Hormone (AMH) (0.54 ng/mL, normal range 0.77–14.5) and inhibin B (14.9 pg/mL, normal range 16.61–278.87) were both low. DM was poorly controlled, with fasting glucose of 188 mg/dL and a HbA1c of 7.5%. Liver enzymes were within normal range.

**Figure 1:**
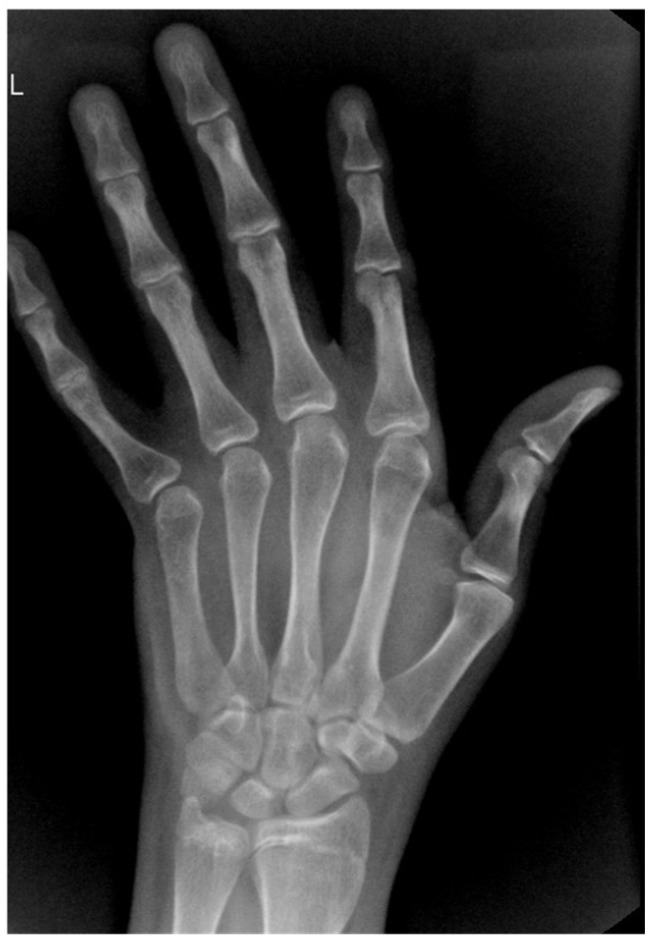
Case 1: left hand X-ray—bone age of 17 years.

Abdominal ultrasound revealed fatty liver. The left-hand showed a bone age of 17 years (Figure 1). Pituitary MRI revealed a partially empty sella with a maximal pituitary height of 4.3 mm, without any abnormality of the neurohypophysis. Pelvic MRI did not detect any testicular structures, seminal vesicles, or components of the spermatic cord. Mullerian structures were absent, and the prostate was hypoplastic.

An old left hip fracture became apparent. Low bone mass was confirmed by Dual X-Ray Absorptiometry, with a lumbar spine Z-score of −3.1 SD and a Neck Z-score of −1 SD.

The suspicion of Marfan syndrome was excluded after ophthalmological and cardiac ultrasound evaluations. Karyotype returned 46, XY.

The patient was started on prednisone 5 mg/day, levothyroxine 75 μg/day, testosterone gel 40 mg/day, alendronate 70 mg/week, and vitamin D 2000 IU/day, with favorable outcomes. Last follow-up at the age of 45 years old showed adequate substitution with 75 µg/day levothyroxine and 5 mg/day prednisone. Testosterone was still low and thus the testosterone gel dose was increased from 40 mg/day to 60 mg/day. Estradiol levels were within normal range (12 pg/mL, NR 7–42) (Table 1).

### 2.2. Case 2

A 17-year-old boy, with a history of operated adamantinomatous craniopharyngioma at the age of 5, for which surgical reintervention was necessary at the ages of 9, 14, and 15, respectively, when gamma-knife was also performed, developed post-operative hypopituitarism, for which hydrocortisone, levothyroxine, and desmopressin were initiated. Low-dose recombinant hGH therapy (1 mg/day, equivalent to 0.01 mg/kg/day) was started at 10 years of age, after 12 months of disease remission and GH deficit confirmation via low IGF-1 and negative glucagon dynamic testing. It was continued until the age of 14, when it was stopped due to tumor recurrence. Testosterone substitution was started at 15 years of age, when he was already 189 cm tall.

**Figure 2:**
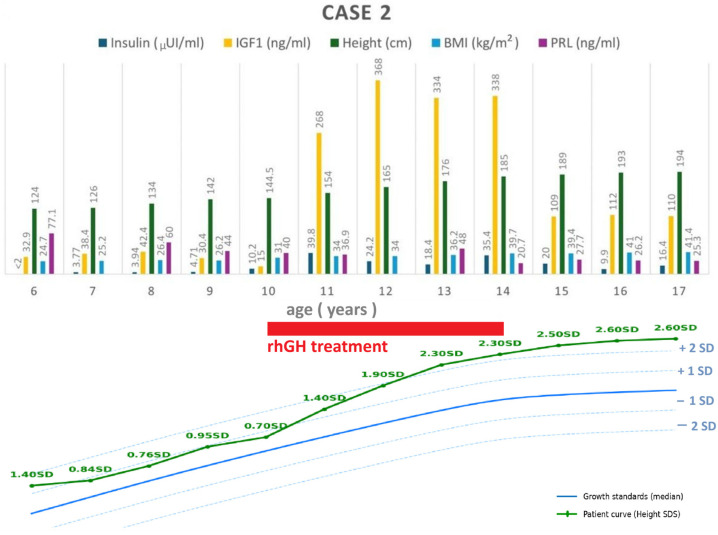
Case 2: longitudinal follow-up of anthropometric parameters, IGF-1 and hormonal profile (**top**), and height standard deviation score compared to growth standards (**bottom**); BMI = body mass index; IGF-1 = insulin-like growth factor 1; rhGH = recombinant human growth hormone; PRL = prolactin; SDS = standard deviation.

Despite the patient having permanently low IGF-1 levels since the diagnosis until GH was initiated and after recombinant hGH therapy was stopped, he continued to grow in both these periods of time (Figure 2). Left hand X-ray performed at the age of 15 (Height = 189 cm, +2.5 SDS) showed fused growth plates, with a bone age of 18 years, but he subsequently gained 5 cm (Figure 2). Residual lower-limb physeal activity likely explains the growth; small measurement variability (due to device, time of day, or posture) may have contributed to it.

**Figure 3:**
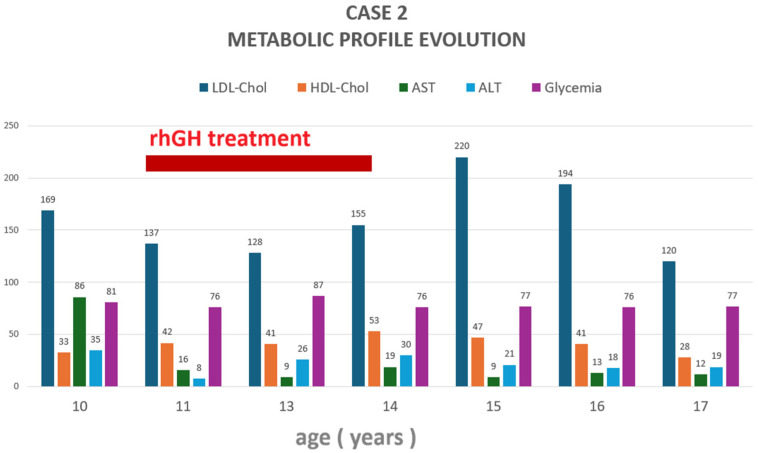
Case 2: evolution of the metabolic profile during follow-up. LDL-Chol = low-density lipoprotein cholesterol (expressed as mg/dL), HDL-Chol = high-density lipoprotein cholesterol (expressed as mg/dL); ALT = alanine aminotransferase (expressed as U/L); AST = aspartate aminotransferase (expressed as U/L); rhGH = recombinant human growth hormone. Glycemia (fasting glucose) is expressed as mg/dL.

Along the course of the disease, he developed hypothalamic syndrome with hyperpyrexia, hyperphagia, progressive hyperinsulinemia, fatty liver (enlarged hypoechoic liver on ultrasound), and obesity. Prolactin levels remained slightly elevated, up to three times the upper limit of normal (Figure 2, Table 1), likely due to the pituitary stalk effect. Dual X-ray absorptiometry (DXA, Hologic Delphi A; Hologic Inc, USA) body composition analysis revealed an increased trunk-to-leg fat ratio of 1.6. Metabolic profile significantly improved during hGH therapy, with hypercholesterolemia reoccurring after cessation (Figure 3).

Clinical examination at the last follow-up visit (17 years of age) revealed tall stature of 194 cm (+2.6 SDS for age and sex), severe obesity (weight = 156 kg, BMI = 42.51 kg/m^2^, >99th percentile), genu varum, and impaired gait.

Lipid profile, fasting glucose, liver enzymes, and renal function were within normal limits. HbA1c was 5.9%, suggesting prediabetes. Hormonal work-up confirmed adequate substitution on the thyrotroph and corticotroph axis. Due to serum testosterone levels at the lower limit under topical testosterone gel 40 mg/day administration, the daily dose was increased to 60 mg/day (Table 1). Recombinant hGH therapy substitution for body composition was reinitiated at a transitional dose of 0.3 mg/day, according to national protocols.

### 2.3. Case 3

**Figure 4:**
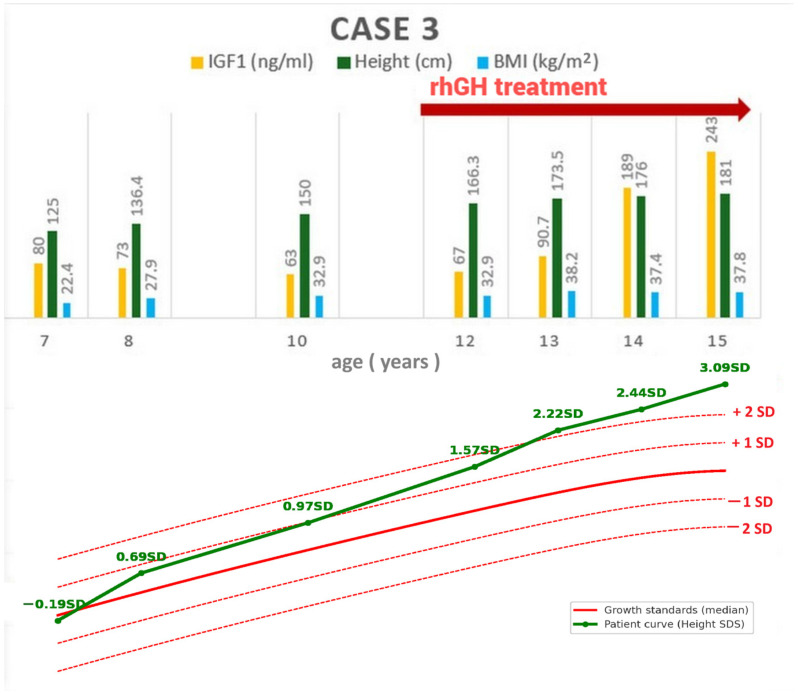
Case 3: longitudinal follow-up of anthropometric parameters and IGF-1 (**top**), and height standard deviation score compared to growth standards (**bottom**). BMI = body mass index; IGF-1 = insulin-like growth factor 1; rhGH = recombinant human growth hormone; SDS = standard deviation.

A 15-year-old female adolescent, with a personal history of adamantinomatous craniopharyngioma for which she underwent surgery at the ages of 7 and 13 years, respectively, developed postsurgical panhypopituitarism for which hydrocortisone, levothyroxine, and desmopressin replacement therapy was initially started. From diagnosis, she had a mean growth velocity of 8 cm/year, with progressive height gain from 125 cm (−0.19 SDS) to 166.3 cm (+1.57 SDS) at age 12 years, despite persistently low IGF-1 levels (Figure 4). GH replacement therapy was started at the age of 12 years old in a standard dose of 0.025 mg/kg/day, reduced after 1 year to a transition dose of 0.5 mg/day, and continued thereafter. Estradiol and progesterone replacement therapy was initiated at the age of 13 years, when she was 173.5 cm tall.

Clinical examination at 15 years of age revealed tall stature (181 cm, +3.09 SDS for age and sex), severe obesity (BMI = 37.8 kg/m; 99th percentile), and a regular menstrual cycle on estrogen-progestin replacement therapy. Hormonal work-up confirmed adequate levothyroxine and glucocorticoid substitution. IGF-1 levels were within normal range under hGH therapy 0.6 mg/day (Table 1). Fasting and 2 h after oral glucose tolerance test revealed normal glycemic values (75 mg/dL and 102 mg/dL, respectively). HOMA-IR index was 3.4 (reference range < 2.4), suggesting insulin resistance. Liver enzymes were within normal range. Fatty liver was confirmed by abdominal ultrasound.

### 2.4. Case 4

A 14-year-old adolescent girl has been followed by our Endocrinology Department since the age of 8 years and 2 months, when she presented with short stature (113 cm, −2.83 SDS for age and sex) and panhypopituitarism secondary to a 56 mm craniopharyngioma, for which she underwent two surgical interventions. At diagnosis, replacement therapy with hydrocortisone, levothyroxine, and desmopressin was initiated.

**Figure 5:**
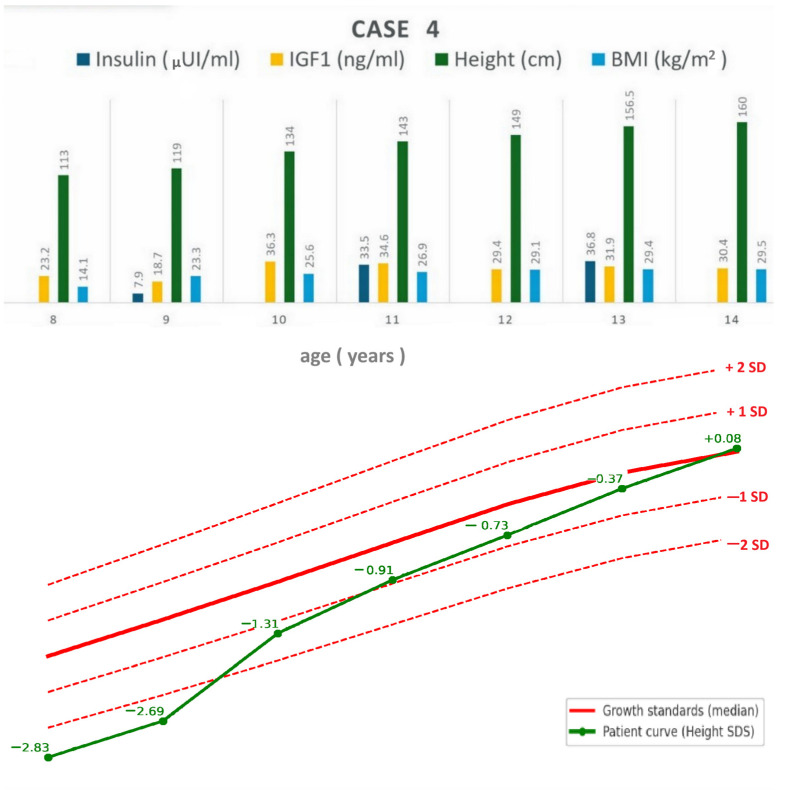
Case 4: longitudinal follow-up of anthropometric parameters and IGF-1 levels (**top**), and height standard deviation score compared to growth standards (**bottom**). BMI = body mass index; IGF-1 = insulin-like growth factor 1; SDS = standard deviation.

During follow-up, the patient developed obesity and hyperinsulinemia and continued to grow, reaching a normal height for age and sex without hGH treatment and despite permanently low IGF-1 levels (Figure 5). At 12 years and 6 months old, when she had a height of 149 cm (−0.73 SDS for age and sex), low-dose estradiol patch therapy was added. Her prolactin levels were permanently low.

At the most recent visit, at 14 years old, she had a height of 160 cm (0 SDS for age and sex, growth velocity up to 7.5/year), weight 75.5 kg, BMI 29.5 kg/m^2^ (96.5th percentile), Tanner pubertal stage P1B3, and axillary and cervical acanthosis nigricans. Laboratory assessment showed adequate hormonal replacement with levothyroxine 100 µg/day, hydrocortisone 10 mg/day, and desmopressin 60 µg twice daily (24 h urine output 2000 mL with normal serum electrolytes) (Table 1). She also presented with biologically confirmed insulin resistance, with a HOMA-IR value of 7.2 (reference range < 2.4). Fatty liver was present on abdominal ultrasound; however, liver enzymes were normal.

### 2.5. Case 5

A 16-year-old female patient with a history of adamantinomatous craniopharyngioma, initially resected at the age of 5, with a second surgical intervention at 7 years old, developed post-surgical panhypopituitarism for which hormone replacement therapy with hydrocortisone, levothyroxine, and desmopressin was started.

**Figure 6:**
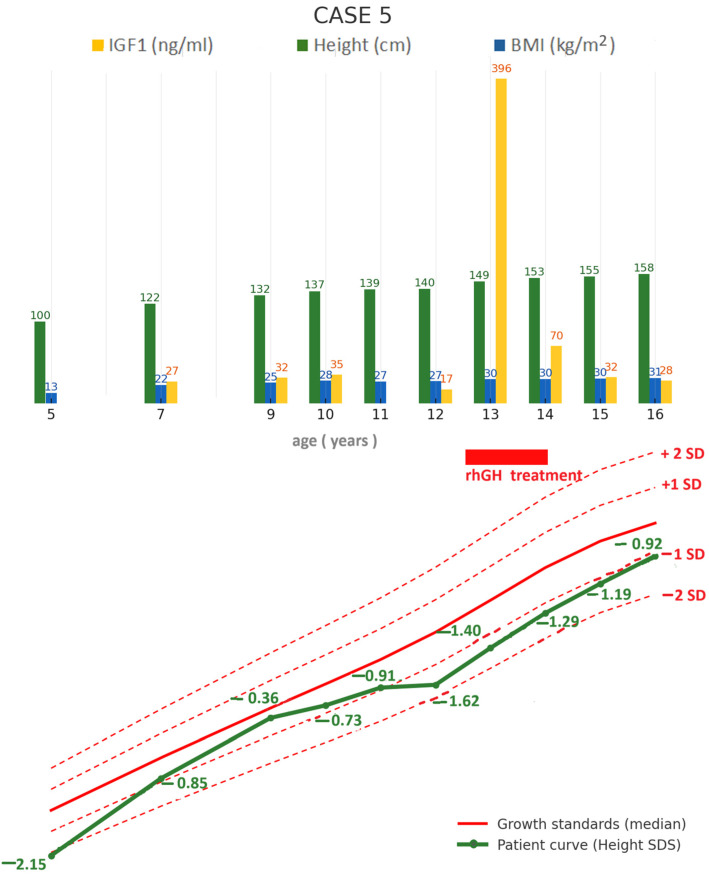
Case 5: longitudinal follow-up of anthropometric parameters and IGF-1 levels (**top**), and height standard deviation score compared to growth standards (**bottom**). BMI = body mass index; IGF-1 = insulin-like growth factor 1; SDS = standard deviation, rhGH = recombinant human growth hormone.

During follow-up, the patient showed satisfactory linear growth despite persistently low IGF-1 levels and the absence of hGH therapy in early childhood, as her weight and BMI continued to increase (Figure 6). Her height increased steadily from 100 cm (−2.15 SDS) at age 5 to 132 cm at age 9 (−0.36 SDS); afterwards her growth velocity gradually declined, and hGH therapy was initiated at 12 years and 6 months when her height was 140 cm (−1.62 SDS). Treatment was discontinued after 14 months due to headaches (Figure 6). Despite stopping hGH, she continued to gain height and progressive weight gain (Figure 6).

**Figure 7:**
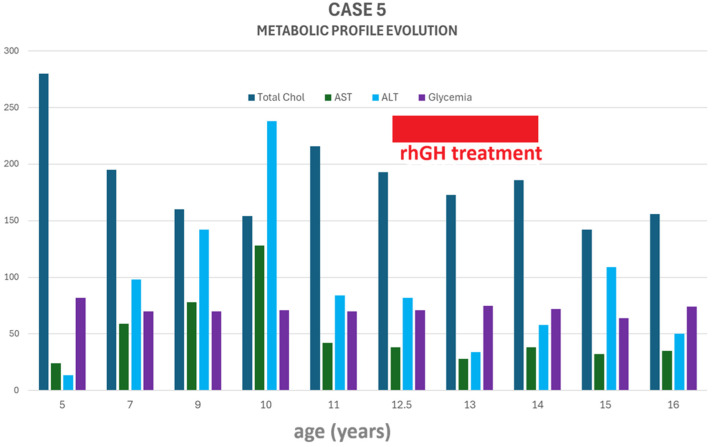
Case 5: evolution of the metabolic profile during follow-up. Total Chol = total cholesterol (expressed as mg/dL), ALT = alanine aminotransferase (expressed as U/L); AST = aspartate aminotransferase (expressed as U/L); rhGH = recombinant human growth hormone. Glycemia (fasting glucose) is expressed as mg/dL.

Over the course of the disease, in addition to developing obesity, she also developed hypercholesterolemia and non-alcoholic steatohepatitis (persistent fatty liver on repeated abdominal ultrasound and elevated liver enzymes). Cholesterol levels slightly improved during hGH therapy and remained within normal limits after treatment discontinuation, despite increasing BMI. In contrast, liver enzymes significantly improved during hGH therapy, with recurrence of transaminitis after treatment cessation (Figure 7).

Pubertal induction with transdermal estradiol began at 15 years, and menarche occurred at 16 years. Prolactin levels remained persistently low throughout follow-up.

At her most recent evaluation at age 16, she presented with a height of 158 cm (−0.92 SDS), a weight of 77.4 kg, and a BMI of 31 kg/m^2^ (97th percentile), with pubertal stage P2B4. Current therapy includes levothyroxine 75 µg/day, hydrocortisone 15 mg/day, and desmopressin 60 µg three times daily, with laboratory assessment confirming adequate hormonal replacement (Table 1).

## 3. Literature Review and Discussion

These five patients illustrate the uncommon but well-documented phenomenon of linear growth despite severe GH deficiency and persistently low circulating IGF-1, highlighting the complexity of growth regulation beyond the classical pituitary GH/IGF-1 axis.

### 3.1. Growth Without GH: Overview and Historical Concept

Normal growth is a multifaceted process influenced by a variety of growth factors and hormones that regulate cellular proliferation, differentiation, and overall physiological development. Key growth factors, such as IGF-1 and IGF-2 and fibroblast growth factors (FGFs), are vital for embryonic and postnatal growth [16,17].

During early childhood, growth is predominantly influenced by nutritional factors and hormonal regulation, with GH and IGF-1 playing vital roles in promoting linear growth and body composition changes. The nutritional status during this period is critical, as deficiencies can lead to impaired growth and long-term developmental delays [1,18,19].

During puberty, sex steroids such as estrogen and testosterone amplify the effects of GH and IGF-1, leading to an acceleration of growth in this developmental phase [20]. Estrogen, particularly, facilitates the growth plate’s activity during early puberty but eventually leads to its closure, marking the termination of growth. In addition to the GH/IGF axis, thyroid hormones stimulate somatic growth by promoting bone development, cartilage maturation, and overall metabolic activity essential for somatic growth. Furthermore, insulin activates intracellular signaling pathways (mTOR, MAPK) that drive protein and cell growth, enhances nutrient uptake, suppresses catabolism, and enables the full action of growth hormone/IGF-1 [21,22]. Prolactin also contributes to growth regulation, although its effects are context-dependent, primarily supporting the development and maintenance of specific tissues [23]. Finally, numerous genetic variants in genes that regulate growth plate chondrogenesis have been uncovered [1].

Growth without growth hormone has been reported for more than 60 years [24,25,26]. Early reports in the 1960s and 1970s described children with craniopharyngioma who exhibited progressive somatic growth despite profound pituitary failure and undetectable GH levels following surgery and radiotherapy [24,25]. At that time, the concept was attributed to an unknown growth-promoting factor capable of stimulating epiphyseal cartilage in a GH-independent manner. With the later discovery and characterization of IGF-1 and IGF-2 and the refinement of hormone assays, it became clear that “growth without GH” was not due to a single hormone substitute, but rather reflected the capacity of multiple metabolic, endocrine, and paracrine pathways to support chondrocyte proliferation and bone elongation. Multiple hypotheses have been further described [26,27,28,29]. Table 2 summarizes published clinical cases describing “growth without GH,” outlining etiologies, metabolic profiles (obesity, hyperinsulinemia, hyperleptinemia), use of hGH, height outcomes, and the hypothesized mechanisms underpinning preserved or accelerated linear growth.

### 3.2. Metabolic Pathways Supporting Growth in GH Deficiency

“Growth without GH” appears particularly common after craniopharyngioma surgery. Multiple case reports describe normal or even accelerated linear growth despite biochemical GH deficiency in this setting, often alongside obesity and hyperinsulinemia (Table 2). Beyond craniopharyngioma survivors, this growth pattern has been documented in congenital hypopituitarism [6], PROP1 mutations [9], septo-optic dysplasia [11,30], and pituitary stalk interruption syndrome [7,8,31], where affected children attained near-normal or tall stature despite persistently low GH and IGF-1 levels [5,6,7,8]. These findings highlighted the contribution of alternative growth-promoting pathways, including hyperinsulinemia, leptin signaling, hyperprolactinemia, sex-steroid–mediated growth plate activation, and adequate circulating thyroid hormones, which together may sustain chondrocyte proliferation and bone elongation when GH action is minimal (Table 2).

First, insulin and metabolic factors can provide anabolic signals to the growth plate and increase IGF-1 bioavailability in GH-deficient children who underwent surgery for craniopharyngioma, as reported by Tiulpakov et al. [32]. All our patients displayed features of obesity and hyperinsulinemia, which are strongly associated with advanced skeletal maturity [40]. The increased fat mass and insulin milieu in our patients likely contributed to the observed growth, as previously suggested in reports of “growth without GH” in children with craniopharyngioma [5,39].

Hyperleptinemia associated with increased fat mass is also thought to promote chondrogenesis. IGF-1 is nutritionally sensitive and may be modulated by leptin, a long-term energy signal, though direct human evidence is scarce. In children with congenital leptin deficiency, leptin replacement—despite weight loss—raised IGF-1 SDS and the IGF-1/IGF-binding protein-3 (IGFBP3) ratio and increased height SDS/growth velocity; these findings suggest leptin directly promotes IGF-1 secretion and linear growth (potentially via hepatic effects, increased GH sensitivity, or GH-independent actions) [41].

Nonetheless, elevated levels of IGF-2 have been documented in a case of accelerated linear growth despite combined pituitary hormone deficiency [38]. IGF-2 is synthesized primarily by the liver, but also by many other tissues, with most of its biological effects occurring after binding to the IGF-1 receptor and insulin receptor A, respectively. IGF-2 expression is tightly controlled by imprinting, promoter usage, IGFBPs, tissue-specific receptor balance and expression, and nutritional–hormonal signals; among the latter, GH has a weak positive effect on IGF-2 expression. Besides its role in embryogenesis, IGF-2 supports postnatal tissue growth and maintenance by promoting angiogenesis, immune-cell and β-cell proliferation, and musculoskeletal development, including potent anabolic and survival effects on osteoblasts. IGF-2 levels are within the normal range in about half of patients with GH deficiency, compared to 82% of these cases having low IGF-1 [42]. Moreover, IGF-2 serum concentrations are elevated in obesity and correlate with BMI [43]. However, studies have failed to prove a direct involvement of IGF-2 in “growth without GH” [11], although elevated serum IGF-2 levels have been reported in an isolated case of idiopathic hypopituitarism that exhibited accelerated growth [38] (Table 2).

Although our cases underscore marked mechanistic heterogeneity, all of them had in common obesity and metabolic complications, such as insulin resistance, dyslipidemia, or fatty liver. Similarly to Case 1, normal or even tall stature attained in the presence of GH deficit in patients with congenital anomalies of the pituitary gland or stalk and even confirmed with PROP1 mutation have also been reported: both in patients with metabolic complications by Lee et al. [8], Makras et al. [37], Den Ouden et al. [13] and Arroyo et al. [9], but also in patients free of obesity or hyperinsulinemia, such as the cases reported by Kageyama et al. [35] or Wu et al. [7]. Obesity with hyperphagia is frequent in children operated for craniopharyngiomas; cases with accelerated growth were reported after surgical intervention, concomitant with weight gain [5,36], similar to cases 2–5 in the current manuscript. Nagasaki et al. [5] also reported sustained “growth without GH” in a pediatric case of Langerhans cell histiocytosis.

Second, high levels of prolactin were long thought to promote bone elongation [26,28]. Prolactin receptor is expressed by the cartilage, while members of the prolactin family (placental lactogen) support fetal growth [44,45]. Evidence for prolactin modulating the human GH/IGF axis is mixed, but animal and in vitro data show prolactin can enhance chondrocyte activity and may increase hepatic IGF-1 signaling [44,46,47].

More so, rising insulin and prolactin may counteract IGFBP3, increasing IGF1 bioavailability and thus enhancing its growth-promoting effects [7]. Early reports of excessive growth showed increased basal prolactin after craniopharyngioma surgery in children with overgrowth, concurrent with normal IGF1 levels [33]. Similarly, GH-deficient patients with prolactin-secreting tumors had IGF-1 levels within the normal range, whereas patients with GH deficiency and normal prolactin had low IGF-1 levels [48].

All the cases reported herein had low or normal prolactin concentrations, except Case 2, which showed mild persistent hyperprolactinemia due to pituitary stalk syndrome. Similarly, most cases reported in the literature had prolactin levels within the normal range [6,7,35,37,39]. Lee et al. [8] and Guo et al. [31] reported mild prolactin elevation in patients with combined pituitary hormone deficiency due to pituitary stalk interruption syndrome and normal growth. Pavlou et al. [36] also reported accelerated growth in a prepubertal boy with mild hyperprolactinemia after removal of a suprasellar craniopharyngioma, similar to our Case 2 [33].

Third, sex steroids—particularly estrogen (via aromatization in boys and replacement in girls with hypopituitarism)—amplify growth plate chondrogenesis and may drive pubertal growth spurts despite low IGF-1 [20,21]. Estrogen is the principal timer of growth-plate maturation and fusion in both sexes. Beyond its indirect stimulation of the GH/IGF-1 axis, estrogen acts locally on chondrocytes (ERα/ERβ) to accelerate hypertrophic differentiation and growth-plate senescence; the cumulative estrogen exposure largely determines the timing of epiphyseal closure [49]. Peripheral aromatization may indeed contribute to growth in obese individuals with limited GH activity. Adipose tissue expresses aromatase, enabling conversion of adrenal androgens to estrogens, which can enhance growth-plate chondrogenesis and promote pubertal growth acceleration even in the setting of low systemic IGF-1. Thus, in obese children, increased peripheral estrogen production may partially compensate for reduced GH action by stimulating epiphyseal cartilage maturation and linear growth [50]. Conditions of chronic hypoestrogenism (congenital/acquired hypogonadism and CYP17A1 defects) in which delayed epiphyseal closure produces disproportionate tall stature are mechanistically distinct from GH but highly relevant [13,14,15,49].

However, Cases 2–5 already exhibited catch-up “growth without GH” when sex hormone substitution was started. Conversely, Case 1, similar to the case reported by Den Ouden et al. [13], had profound lifelong hypogonadism with absent testes, resulting in hypoestrogenism and delayed epiphyseal fusion, a well-recognized route to prolonged, slow linear growth. Even with low IGF-1, GH-independent drivers—notably hyperinsulinemia in our case may have sustained chondrogenesis at low rates over many years; in the absence of sex steroids, epiphyseal fusion is postponed, allowing eventual near-normal/tall-normal adult height [1]. However, very low circulating levels of GH in early and late childhood cannot be excluded in this case.

Fourth, adequate thyroid hormone replacement in Cases 2–5 provided the permissive metabolic environment necessary for growth plate activity; appropriate glucocorticoid replacement avoided catabolic suppression.

### 3.3. Genetic and Local Growth Plate Mechanisms

Tall stature without GH can result from genetic variants that boost growth-plate output or delay epiphyseal fusion—e.g., activating natriuretic peptide receptor 2 (*NPR2*) signaling or *NPR3* loss, short stature homeobox gene (SHOX) duplication, fibrilin 1 (*FBN1*) mutations, CYP19A1 (aromatase) defects, or overgrowth-syndrome genes such as nuclear receptor binding SET domain protein 1 (*NSD1*) or enhancer of zeste homolog 2 (*EZH2*) [12,51,52,53,54,55]—driving GH-independent chondrogenesis. Aromatase deficiency, leading to delayed epiphyseal closure and tall stature, is unlikely in our Case 1, given the profoundly low testosterone and absent testes, and also increasing serum estradiol levels after topical testosterone administration; lifelong hypogonadism with hypoestrogenism—and thus delayed growth-plate fusion makes a better explanation for Case 1. It should be noted, however, that measurements of serum estradiol in men and boys using conventional immunoassays may lack accuracy, particularly at low concentrations, as these assays often overestimate true values; liquid chromatography–tandem mass spectrometry (LC–MS/MS) provides greater specificity in this context [56].

Bereket et al. [30] and Hathout et al. [11] reported normal growth in two patients with septo-optic dysplasia despite profound GH/IGF-1 axis impairment and no GH substitution therapy; in both, growth was not attributable to IGF-1, IGF-2, insulin, or leptin, again pointing beyond canonical pathways [11,30]. Later, El Kholy et al. [10] reported a heterozygous homeobox ES X-linked 1 gene (*HESX1*) missense (Q6H) in a case of combined pituitary hormone deficiency with normal BMI and insulin sensitivity that also showed normal adult height; exome data revealed additional heterozygous variants in signaling genes (Table 2) with variable penetrance, suggesting polygenic modulation of growth signaling that could bypass the GH-IGF-1/insulin axis [10] (Table 2).

Finally, local paracrine mechanisms involving the growth plate (e.g., IGF-2, Indian hedgehog/parathyroid hormone-related protein (IHH/PTHrP), FGFs, etc.) [52] can sustain chondrogenesis when GH/IGF-1 is limited, offering an additional explanation for preserved growth velocities [44].

### 3.4. Metabolic Risk and GH Therapy Considerations

Most patients with “growth without GH” reported in the literature had metabolic disturbances, such as obesity, dyslipidemia, or fatty liver disease (Table 2), including cases reported in this manuscript.

While GH replacement therapy in adult patients with panhypopituitarism improves body composition and fatty liver disease [57,58], the long-term effects of its use for this purpose in children and adolescents with “growth without GH” is not fully understood; Schoenle et al. [34] and Nagasaki et al. [5] reported improved lipid profile, BMI, visceral fat and adipokine profile in such cases treated with hGH for 12 months, without major changes in growth velocity. Pavlou et al. [36] also reported normalization of renal phosphate handling during a 4-day trial of hGH administration in a case of post-surgical panhypopituitarism with accelerated growth. We reported three patients who underwent hGH therapy for 4 years (Case 2, low-dose hGH), 3 years (Case 3, low-dose hGH), and 14 months (Case 5, standard dose), respectively. Case 2 experienced lipid profile improvement, such as increases in LDL-cholesterol and decreases in HDL-cholesterol values, respectively, after hGH initiation, with recurrence of severe dyslipidemia upon treatment cessation, underpinning the well-known beneficial metabolic effects of GH replacement. Serum transaminases were mildly elevated before hGH initiation, but normalized and remained normal thereafter, suggesting an intercurrent/temporary cause. Height velocity remained within the 9–11 cm/year trend during treatment. Unfortunately, a complete metabolic profile history was not available in Case 3; nevertheless, her growth rate did not seem to be influenced, as it progressively diminished under hGH therapy, probably due to concurrent estradiol initiation. Case 5 also received a short course of hGH therapy at standard doses; however, her growth trajectory continued to progress regardless of stopping treatment. She demonstrated marked improvement in liver enzyme levels during hGH therapy, followed by recurrence of transaminitis after discontinuing hGH. Her hypercholesterolemia gradually improved, beginning shortly after hGH initiation, and became even more pronounced after therapy cessation. All three patients treated with hGH maintained normal fasting glucose levels throughout the treatment period. Thus, hGH may have a more pronounced effect on body composition, lipid profiles, and steatohepatitis than on height velocity when baseline linear growth is already adequate.

### 3.5. Bone Health Considerations

Although obesity is generally associated with higher areal BMD via increased mechanical loading that favors bone formation, metabolic and inflammatory disturbances associated with excessive adiposity can impair bone quality and microarchitecture, contributing to increased risk of peripheral fractures [59]. More so, visceral adiposity is associated with lower areal BMD Z-scores in overweight and obese children [60]. Areal BMD measurement can also be confounded by large body size, excess fat and lean mass, positioning difficulties, and greater precision error in obese children [61]. GH deficiency and hypogonadism may further hinder bone mass acquisition during adolescence. A history (or absence) of fracture remains an important adjunct to densitometry [59,61]. In our series, only Case 1 had a documented prior fracture, due to osteoporosis secondary to prolonged hypogonadism; no other fractures were identified. Nevertheless, DXA interpretation in these patients requires caution, as increased adiposity may falsely elevate Z-scores, potentially masking underlying skeletal vulnerability [61].

### 3.6. Clinical Implications

These cases, together with accumulating evidence, support a paradigm shift: GH deficiency does not always manifest with impaired linear growth, particularly in the setting of hypothalamic obesity and altered metabolic signaling. In such individuals, preserved or accelerated stature should not delay diagnosis or treatment of GH deficiency or other pituitary deficits. Instead, clinical priorities should emphasize [1] cardiometabolic protection (dyslipidemia, insulin resistance, metabolic dysfunction-associated steatohepatitis) [2], bone health maintenance, especially in those with delayed puberty or hypogonadism, and [3] long-term surveillance into adulthood, given the persistent metabolic vulnerability.

In selected patients, hGH therapy may be considered primarily for metabolic indications rather than height optimization, particularly in the presence of central adiposity, dyslipidemia, or evolving fatty liver disease. Treatment decisions should remain individualized, with careful monitoring of body composition, glucose–insulin dynamics, and lipid profiles.

In conclusion, these cases highlight that near-normal or even accelerated linear growth can occur despite profound GH deficiency and very low circulating IGF-1, underscoring the intricate interplay of metabolic, endocrine, and local growth-plate pathways that can compensate for absent GH action. The common thread across our cohort was metabolic vulnerability, characterized by obesity, insulin resistance, fatty liver, and dyslipidemia—features often seen in hypothalamic dysfunction and central adiposity-driven growth patterns. Notably, hGH therapy improved lipid profile and steatohepatitis in two of the patients reported. Recognition of “growth without GH” should prompt clinicians to look beyond height velocity and target broader health outcomes. Proactive metabolic risk reduction, timely hormone replacement, and lifelong follow-up from childhood into adulthood are essential to optimize long-term cardiometabolic health and quality of life in this unique population.

## Figures and Tables

**Table 1:** Growth-related clinical and hormonal parameters in the five cases.

Work-Up	Case 1	Case 2	Case 3	Case 4	Case 5
Diagnosis					
Age (y)	43	5	7	8	5
H (cm)	182.5	117	125	113	100
W (kg)	81	31	35	18.5	13
BMI (kg/m^2^)	24.5 kg/m^2^	22.6 (>99th percentile)	22.4 (9th percentile)	14.1 (19th percentile)	13 (<1st percentile)
Follow-up period	2 y	12 y	8 y	6 y	12 y
hGH treatment	No	Yes, for 4 years	Yes, for 3 years	No	Yes, for 14 months
Last follow-up visit ^#^					
Age (y)	45	17	15	14	16
H (cm)	182.5	194	181	160	158
W (kg)	83	156	124	75.5	77.4
BMI (kg/m^2^)	25	42.5 (>99th percentile)	37.8 (99th percentile)	29.5 (96.5 percentile)	31 (97th percentile)
IGF-1 (ng/mL)	<15	110 (162–584)	243 (220–972)	30.4 (220–972)	27.7 (226–903)
FT4 (0.89–1.76 ng/dL) *	1.13	1.45	1.18	1.13	1.14
Prolactin (ng/mL)	6 (NR 1.5–17)	25.3 (NR 1.5–17)	12 (NR 1.9–25)	1.5 (NR 1.9–25)	6.05 (NR 1.9–25)
E2 (pg/mL)/T (nmol/L) **	T = 6.5 nmol/L (8.64–2.9)	T = 8.92 nmol/L (3.4–41)	E2 = 26 pg/mL (5–68)	E2 = 25 pg/mL (5–68)	E2 = 30 pg/mL (5–68)

Note: ^#^ all patients were under glucocorticoid substitution therapy, therefore cortisol levels were not informative and were not monitored; * under LT4 substitution therapy; ** under topical testosterone/estradiol substitution therapy. BMI = body mass index; E2 = estradiol; FT4 = free thyroxine; hGH = human growth hormone; H = height; IGF-1 = insulin-like growth factor 1; NR = normal range; T = testosterone; W = weight.

**Table 2:** Published clinical cases describing “growth without GH,” outlining etiologies, metabolic profiles, use of hGH, height outcomes, and potential explanations for this phenotype.

Article	Number of Patients (N)	Etiology of GH Deficiency	Obesity/Hyperinsulinemia/ Hyperleptinemia	hGH Therapy	Final Height	Possible Explanation
Bereket et al. (1998) [30]	N = 1	Septo-optic dysplasia	Obesity Hyperinsulinemia	No	Normal growth velocity	Unexplained growth not mediated by IGF-1 or insulin
Guo et al. (2024) [31]	N = 24	Pituitary stalk interruption syndrome	Normal BMI	No hGH therapy in GH-deficient patients exhibiting normal growth	N = 4 with normal adult height without hGH therapy N = 8 short stature without hGH therapy N = 12 with a history of hGH therapy	Delayed epiphyseal fusion (hypogonadism) and mild hyperprolactinemia
Tiulpakov et al. (1988) [32]	N = 25	Postsurgical GH deficiency (craniopharyngioma)	Obesity/hyperinsulinemia	No	Normal growth velocity (N = 4) Reduced growth velocity (N = 21)	Insulin increases IGF-1 bioavailability
Holmes et al. (1968) [24]	N = 9	Postsurgical GH deficiency (craniopharyngioma—N = 8, third ventricle cyst—N = 1)	Frequently overweight; hyperinsulinemia discussed	No	Normal growth and adult height	Insulin/IGF-like circulating activity; GH-independent growth; delayed epiphyseal fusion in hypogonadism
Costin et al. (1976) [25]	N = 8	Postsurgical panhypopituitarism (craniopharyngioma)	Obesity (n = 5) Hyperinsulinemia/higher than expected insulin levels (N = 7)	No	Normal postoperative growth (5 cm/year) for various periods of time	Insulin potentiating somatic growth; increased IGF bioactivity
Bucher et al. (1983) [33]	N = 19	Postsurgical GH deficiency (craniopharyngioma)	Many obese/hyperinsulinemia	No	Mixed: excessive/normal growth (N = 13) Reduced growth (N = 6)	Hyperinsulinemia and normal IGF1-1 in excessive growth. Hyperprolactinemia and normal IGF-1 in normal growth
Geffner et al. (1986) [27]	N = 1	Idiopathic	Normal insulin response to glucose	No	Increased growth velocity	Potent circulating growth factor, distinct from GH and IGF-1
Bistritzer et al. (1988) [26]	N = 3	Not mentioned	Absent	No	Normal growth rate	Unknown bioactive molecule with GH receptor binding affinity
Schoenle et al. (1995) [34]	N = 6	Postsurgical panhypopituitarism (craniopharyngioma)	Overweight/obese	Yes, for 12 months (N = 6)	Normal growth maintained pre-GH therapy with no further increase in height velocity during treatment. Decreased BMI and skinfold thickness and increased calf circumference during hGH therapy	Maintained growth velocity unexplained
Kageyama et al. (1998) [35]	N = 1	Congenital hypopituitarism	Normal body weight Insulin not measured	No	Tall stature	Several growth factors independent of the GH-IGF-I axis not yet identified. Delayed epiphyseal closure (hypogonadism)
Hathout et al. (1999) [11]	N = 1	Septo-optic dysplasia	No evidence of hyperinsulinemia Marked hyperleptinemia	No	Normal linear growth	Bone growth is not mediated by IGF-1, IGF-2, leptin, or insulin. Unidentified growth factor or mechanism
Pavlou et al. (2001) [36]	N = 1	Postsurgical panhypopituitarism (craniopharyngioma)	Obesity with central fat distribution Hyperinsulinemia	Not on long-term (4-day trial)	Accelerated height velocity	Hyperinsulinemia and mild hyperprolactinemia. Renal phosphate handling normalized during the 4-day hGH administration
Arroyo et al. (2002) [9]	N = 1	PROP1 mutation	Obesity Hyperinsulinemia	No	Normal adult height	Low levels of circulating GH in infancy. Delayed epiphyseal fusion from hypogonadism
Den Ouden et al. (2002) [13]	N = 1	Congenital (agenesis of the pituitary stalk)	Overweight (central fat deposition)	No	Tall adult height	Greater than expected effect of low IGF-1 levels in the setting of hypoestrogenism. Increased insulin levels (not proven). Extended growth period (hypogonadism)
Lazar et al. (2003) [6]	N = 5	Idiopathic combined pituitary hormone deficiency	Normal body weight	No	Final height within target height (N = 4) or higher (N = 1)	Extended growth period (hypogonadism). Unknown growth factors
Makras et al. (2004) [37]	N = 1	Hypothalamic hamartoma with panhypopituitarism and empty sella on follow-up	Obesity and hyperinsulinemia	No	Normal height within target, but still growing (open epiphyses)	Hyperinsulinemia
Lee KJ et al. (2009) [38]		Idiopathic	Normal BMI. Insulin resistance		Accelerated growth, reaching mid-parental height	Hyperinsulinemia and delayed fusion of epiphyseal plates (hypogonadism). Elevated IGF-2 levels
Nagasaki et al. (2010) [5]	N = 2	Post-therapy for brain tumors (intracranial Langerhans histiocytosis that underwent chemotherapy, hypothalamic lesion with empty sella—N = 1, craniopharyngioma that underwent surgery and radiotherapy—N = 1)	Increased body fat Obesity Hyperinsulinemia Hyperleptinemia	Yes, for 12 months	Near-normal growth rate pre-treatment; growth velocity remained stable in the first case but increased in the second case during rhGH therapy. Improvement in transaminitis, decreased triglycerides, and visceral fat during GH replacement therapy	Leptin, insulin, and/or other GH-independent factors
Atay et al. (2015) [39]	N = 1	Postsurgical panhypopituitarism (craniopharyngioma)	Obesity with hyperinsulinemia	No	Final height within normal range	Insulin-mediated growth; normal GH bioactivity despite deficient immunoreactivity or residual GH secretion despite provocative testing
Lee SS et al. (2017) [8]	N = 1	CPHD due to pituitary stalk interruption syndrome	Normal BMI (Truncal adiposity) Normal insulin concentrations Mild hyperleptinemia	No	Normal height (above target height)	Mild hyperprolactinemia
El Kholy et al. (2019) [10]	N = 1	Heterozygous missense HESX1 mutation (Q6H substitution)	Normal BMI Normal insulin sensitivity	No	Normal height	Growth not mediated by IGF-1 or insulin. Heterozygous missense mutations of various genes involved in intracellular signaling pathways (*AKAP12*, *ARAP1*, *SH2B3*, *CDKN2A*, *GPR98*, *IGF2BP3*, *PIK3C2G)* + variable penetrance of the heterozygous missense mutation
Wu & Xu (2024) [7]	N = 1	CPHD due to pituitary stalk interruption syndrome	Normal body weight	No	Tall stature	Low IGFBP-3, possibly increasing the bioavailability of IGF-1. The patient also associated with consanguinity and a mosaic monosomy X karyotype

Note: hGH = human growth hormone, BMI = body mass index, IGF = insulin-like growth factor, *HESX1* = homeobox ES X-linked 1 gene, *AKAP12* = A-kinase anchoring protein gene, *ARAP1* = Arf GAP with Rho GTPase activity gene, *SH2B3* = SH2 adaptor protein 3 gene, *CDKN2A* = cyclin-dependent kinase inhibitor 2A gene, *GPR98* = adhesion G-protein–coupled receptor V1 gene, *IGF2BP3* = insulin-like growth factor 2 mRNA-binding protein 3 gene, *PIK3C2G* = phosphatidylinositol-4-phosphate 3-kinase, catalytic subunit type 2 gamma gene.

## Data Availability

The original contributions presented in the study are included in the article. Further inquiries can be directed to the corresponding author.
